# A Rare Case of Gastric Intramural Hematoma Secondary to Hemorrhagic Pancreatitis

**DOI:** 10.7759/cureus.45039

**Published:** 2023-09-11

**Authors:** Joey Almaguer, Dylan Murray, Sheedeh Motamedi, Richard Murray

**Affiliations:** 1 Radiology, Texas Tech University Health Sciences Center, Amarillo, USA; 2 Surgery, University College Dublin, Dublin, IRL; 3 Radiology, Texas Tech University Health Sciences Center, Lubbock, USA; 4 Diagnostic and Interventional Radiology, Texas Tech University Health Sciences Center, Amarillo, USA

**Keywords:** conservative treatment, diagnostic laparoscopy, hemoperitoneum, lipase, pancreatitis, gastrointestinal hemorrhage, gastric intramural hematoma

## Abstract

Gastric intramural hematoma (GIH) is a contained hemorrhage located within the layers that comprise the wall of the stomach. It is a rare condition that has a variety of etiologies. Pancreatitis-induced GIH is an even rarer phenomenon, with only a handful of documented cases in the medical literature. In the current case, a patient presented with chronic abdominal pain for the past two months, with an acute worsening of symptoms. CT imaging confirmed a large, stable GIH with concomitant pancreatitis, likely alcohol-induced. Diagnostic laparoscopy was performed in response to worsening hemodynamic status, which confirmed hemorrhagic pancreatitis as the likely cause of the GIH formation. Jackson-Pratt (JP) drains were placed, and the patient was subsequently discharged. The patient returned one month later with an acute exacerbation of pancreatitis; however, interval improvement of the GIH was observed. The patient was transferred to outpatient care for continued conservative treatment without any further return visits.

## Introduction

An intramural hematoma is a contained blood collection within the wall of a bodily structure, usually seen in the context of vascular pathology [1]. By extension, a gastric intramural hematoma (GIH) consists of blood collection in between the layers that constitute the wall of the stomach [2]. The condition is rare, with the majority of upper gastrointestinal hematomas being localized to the esophagus or duodenum [3]. A proposed mechanism for the formation of a GIH is the shearing of the terminal arteries within the muscular layer of the gastric wall, leading to hemorrhaging and subsequent separation of the submucosa from the muscularis externa [4]. The causes of a GIH can be external trauma, iatrogenic injury, intestinal pathology (such as a peptic ulcer disease), anticoagulant therapy, coagulopathies, cancers, parasitic infection, pancreatitis, idiopathic, and others [5].

There have been several reported cases of endoscopic procedures that disrupt the gastric wall integrity, inadvertently resulting in a GIH. Percutaneous endoscopic gastrostomy tube placement at the site of the needle insertion has been documented to be one particular cause [6]. Similarly, there have been multiple cases of endoscopic submucosal dissection or resection being another reported source of GIH development [7,8]. Endoscopic ultrasound-guided fine-needle biopsy has been seen to cause a GIH at the site of the needle puncture [9]. Therapeutic interventions, such as epinephrine injections to prevent further bleeding from a hemorrhaging gastric ulcer as well as lesion ablation with argon plasma coagulation, can also cause an inadvertent GIH formation [10,11]. These cases of iatrogenic injury are likely more common in those patients who are medically anticoagulated but can also occur in those with a normal coagulation profile [12].

Hematopathologies, vasculopathies, and medical anticoagulation have been seen to contribute to the formation of a GIH. One case described post-traumatic GIH formation in a patient who was on anticoagulation [13]. Even in patients without associated trauma, multiple cases have described GIH formation in the setting of anticoagulant therapy [14,15]. Bleeding disorders, such as hemophilia, have been discovered after a presenting case of GIH [16,17]. It may also be possible that a combination of both hematopathology and medical anticoagulation could have a synergistic effect on GIH formation such as in a patient with systemic lupus erythematosus on Warfarin therapy [18].

Infections and cancers have also been reported to be rare causes of GIH. One case described a parasitic infection with Anisakis as a cause of GIH development [19]. Acute lymphoblastic leukemia has also been associated with GIH development, possibly due to changes in coagulation [20]. It is important to not mistake a GIH for a gastric neoplasm, such as a gastrointestinal stromal tumor (GIST), as they can appear similarly on radiographic imaging and are the most common mesenchymal tumors of the gastrointestinal tract [21-24]. Similarly, a GIH can also appear similarly to an aneurysm located in the branch of the superior mesenteric artery, which can cause further diagnostic confusion [25].

GIH has also been known to occur spontaneously and without a specific or clear etiology [26-28]. Complications include gastric outlet obstruction, as the hematoma imparts a mass effect on the pyloric sphincter, preventing physiologic evacuation of gastric contents into the small intestines. As previously noted, gastric ulcers can lead to GIH formation. However, it has been shown that duodenal ulcers can also lead to a GIH and subsequent gastric outlet obstruction [29]. The following presented case describes a patient with worsening chronic abdominal pain who was diagnosed with a GIH.

## Case presentation

A 65-year-old male presented to the emergency department complaining of abdominal pain localized to the epigastric area. The pain had been present for the previous two months but had acutely worsened over the past two days. The abdominal pain was accompanied by early satiety, weight loss, and associated nausea. He stated that the pain was worse when he ate and drank, causing him to have less oral intake. His diet consisted mostly of popsicles and alcohol, and he admitted to alcohol use every other day because it helped with his pain. The patient also described episodes of weakness and difficulty walking two to three months prior to episodes of falling, although further details regarding the falls were not obtained nor was a medical evaluation received after the incidents.

The patient had not taken any prescription or over-the-counter medication to address his symptoms. He denied fever, chills, hematemesis, hematochezia, melena, or any change in bowel habits. His past surgical history consisted of a hiatal hernia repair, although the date of this surgery was not specified. On physical examination, there was tenderness to palpation of the epigastric region with no obvious masses palpated. Basic laboratory work-up was remarkable for a Hgb of 12.8 gm/dL, Hct of 38%, and lipase level of 1,638 units/L (Table 1).

**Table 1:** Changes in laboratory values across the duration of the GIH episode The patient’s initial visit was highlighted by a marked elevation in lipase levels with a normal hemodynamic status, reflecting a stable and contained GIH. Hemodynamic status began to fall, with the lowest value being recorded on 09/15/2021. Although lipase levels were improving during this time, the decrease in hemoglobin levels likely reflects the worsening hemorrhagic status of the existing pancreatitis. Increased lipase levels on the one-month return likely reflect chronic alcohol-induced pancreatitis. Amylase levels were not recorded during this time period. GIH: gastric intramural hematoma; Hgb: hemoglobin, Hct: hematocrit

	Hgb (mg/dL)	Hct (%)	Lipase (units/L)
09/11/2021	12.8	38	1,638
09/12/2021	12.1	34	595
09/13/2021	11.8	35	-
09/14/2021	11.2	33	487
09/15/2021	9.5	27	-
09/16/2021	11.4	34	100
10/12/2021	16.7	52	1,059

**Figure 1:**
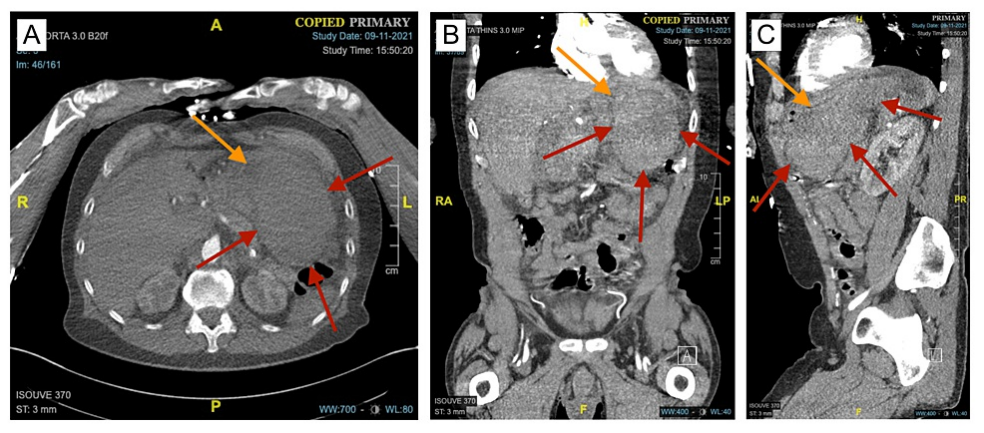
CT angiogram revealing a GIH A, B, C: Axial, coronal, and sagittal views of the initial presentation of the GIH (red arrows). The GIH was imparting a mass effect on the stomach and surrounding structures, compressing the gastric lumen (orange arrows). GIH: gastric intramural hematoma

The patient underwent his first CT of the abdomen and pelvis with IV contrast of the encounter, which revealed a large hyperdense lesion in the left upper quadrant of the abdomen that measured approximately 13 x 8 x 10 cm (Figure 1). The lesion appeared to compress the stomach and measured 57 Hounsfield units, which was consistent with blood. Although the lesion was concerning for a large hematoma, an underlying GIST tumor could not be excluded, as there was no contrast extravasation present. The lesion was accompanied by mild intraabdominal and pelvic ascites consistent with hemoperitoneum.

**Figure 2:**
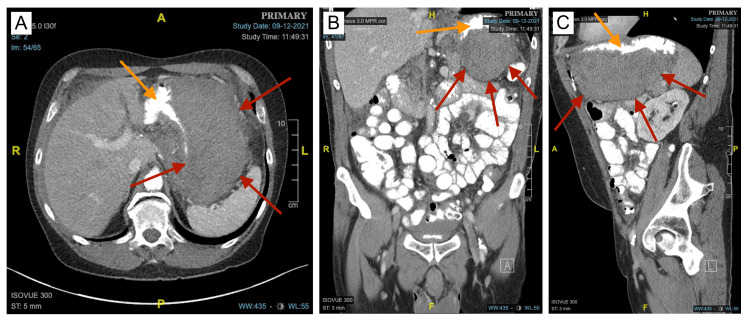
CT of the abdomen and pelvis with IV and oral contrast revealing the GIH A, B, C: Axial, coronal, and sagittal views with oral contrast better illustrate the margins of the GIH (red arrows). Compression of the gastric lumen (orange arrows) due to mass effect from the GIH is better observed. GIH: gastric intramural hematoma

Due to possible intraabdominal bleeding with unspecified etiology, the surgical team was consulted and the patient was admitted for further evaluation and management. Morphine was administered to the patient, which greatly improved his abdominal pain. Repeat laboratory workup was remarkable for Hgb of 11.8 gm/dL, Hct of 35%, and a lipase level of 595 units/L. Repeat CT of the abdomen and pelvis with intravenous and oral contrast showed some increased soft tissue attenuation that extends toward the gastric antrum and first segment of the duodenum with associated mass effect and surrounding mesenteric stranding. There was also hypoattenuation of the uncinate process of the pancreas with the body and tail being unremarkable (Figure 2).

The patient was then consulted by gastroenterology and underwent esophagogastroduodenoscopy, which did not reveal any intraluminal pathology outside of moderate erythema. There was no evidence of any submucosal mass to suggest leiomyoma or gastric GIST. Laboratory levels continued to decrease, with a Hgb of 11.2 gm/dL, Hct of 33%, and lipase of 487 units/L. AFP (alpha-fetoprotein), CA 19-9, and CEA levels were all within normal limits.

The next day, repeat laboratory values showed an Hgb of 9.5 gm/dL, Hct of 27%, and lipase of 487 units/L. Due to worsening hemodynamic status, two units of packed red blood cells (PRBCs) were transfused to the patient. A diagnostic laparoscopy was then performed with suctioning of 700 cc of the intraabdominal hematoma and placement of two JP drainage catheters (one in the pelvis and another in the lesser sac). Fluid lipase levels in one of the JP drains were too high to measure (> 30,000 units/L), which was consistent with hemorrhagic pancreatitis. Given previous CT imaging showing hypoattenuation of the uncinate process of the pancreas, the hemorrhagic pancreatitis was likely chronic in nature. The culture of the serosanguinous abdominal fluid did not result in any microbial growth and the Gram stain was negative. Based on the improving blood lipase levels, the severity of the acute-on-chronic episode of pancreatitis appeared to be improving and was managed conservatively. Because of improved laboratory values, the pelvic JP drain was removed, and the patient was discharged with instructions to keep the lesser sac JP drain in place until the lipase levels within the drain had normalized.

**Figure 3:**
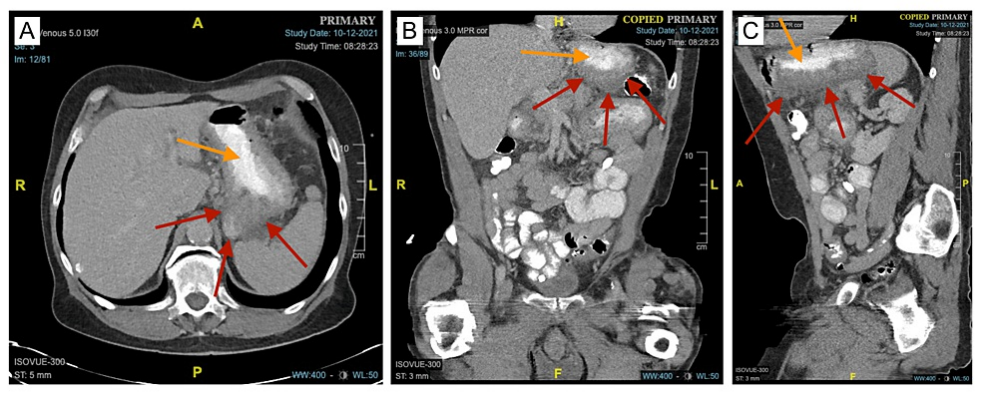
CT of the abdomen and pelvis with oral contrast one month after the initial discharge A, B, C: Axial, coronal, and sagittal views showing interval improvement and a decrease in the overall size of the GIH (red arrows). Due to the reduced size of the GIH, the lumen of the stomach (orange arrows) is less compressed. GIH: gastric intramural hematoma

With the lesser sac JP drain still in place, the patient returned one month later, complaining of severe abdominal pain and increased blood accumulation in the remaining drain. On physical examination, the patient had severe generalized abdominal tenderness. Laboratory values reflected a Hgb of 16.7 gm/dL, Hct of 52%, and lipase of 1,059 units/L. CT of the abdomen and pelvis with contrast noted interval improvement in the soft tissue attenuation, although the soft tissue attenuation extending toward the gastric antrum and first segment of the duodenum still remained. The surrounding inflammatory stranding around most of the head and the uncinate process of the pancreas also remained (Figure 3). Because his condition was stable, he was transitioned to outpatient management and given anticipatory guidance to return if his symptoms worsened. As of two years later, he has not returned for any medical indication.

## Discussion

GIH is a rare occurrence in and of itself, with a reported number of 26 cases documented in the literature as of 2009 [5]. GIH secondary to pancreatitis is an ever-rarer phenomenon with even fewer published papers in the medical literature. Because of this, existing reports on the association between a GIH and episodes of pancreatitis are very limited. Pancreatitis is not the only pancreatic source of GIH formation, as heterotopic pancreatic tissue found within the stomach is also a documented cause of GIH [30]. As far as our search can reveal, there are only five cases of GIH found to be a direct result of pre-existing pancreatitis.

One report postulates that pancreatitis can cause a GIH by way of two mechanisms: pancreatic enzymes causing an irritative effect on the surrounding vascular structures or peripancreatic fluid collections imparting compressive effects on adjacent structures [31]. Similarly, two other reports propose that inflammation from the pancreas can extend into the stomach and erode through the gastric wall, resulting in leakage of pancreatic enzymes that degrade the feeding gastric vessels [32,33]. One report describes a patient with hemorrhagic shock from a persistently bleeding GIH that formed as a result of alcohol-induced necrotizing pancreatitis [34]. Lastly, a pediatric patient was described to have suffered from a GIH as a result of post-endoscopic retrograde cholangiopancreatography (ERCP) pancreatitis causing pancreatitis-induced gastric vessel injury [35].

Although there are no established guidelines for the treatment of a GIH, most of the cases have been treated in a variety of ways, depending on the presumed cause, severity of the condition, and clinical outlook of the patient. Previous cases of GIH have been treated by methods that range from entirely noninvasive to largely invasive surgical intervention. It is generally thought that if the patient is clinically well and hemodynamically stable, the GIH is likely contained, requiring only conservative treatment with nasogastric decompression, intravenous fluid resuscitation, and bowel rest [32]. However, if the patient is decompensating, vitals are unstable, laboratory values reflect hemorrhagic shock, or CT imaging depicts continued contrast extravasation, then more invasive approaches, such as incision and drainage, trans-arterial embolization, or partial gastrectomy, can be considered, depending on the clinical picture [11,36].

Even though the presented patient did admit to suffering from falls, the anatomical location of the impact was not conveyed or documented. Additionally, an acute exacerbation of the condition two months later would not have likely occurred in the setting of trauma. Although trauma-induced GIH formation cannot be entirely ruled out, the concomitant excessive alcohol intake in the setting of markedly elevated abdominal fluid lipase levels makes pancreatitis-induced GIH the most plausible clinical scenario. It is theoretically possible for an existing GIH to impart such a significant mass effect on the surrounding structures that obstructive pancreatitis then manifests, however, our review did not identify such a case being documented in the medical literature. Additionally, if obstructive pancreatitis was present, we would expect to see a resolution of the pancreatitis upon improvement of the GIH. Instead, we observed the opposite: acute worsening of the pancreatitis (likely due to continued alcohol use) with concurrent improvement of the GIH. Given that the patient admits to chronic alcohol intake and that the lipase levels remained elevated even after interval improvement of the GIH, chronic pancreatitis-induced GIH is the most likely clinical diagnosis.

## Conclusions

GIH is an uncommon condition that has a variety of different etiologies and approaches to treatment. Although exceedingly rare, pancreatitis-induced GIH is a documented condition that can occur in both adult and pediatric populations. There are no established guidelines for the treatment of pancreatitis-induced GIH. However, conservative treatment targeted at reducing the severity of pancreatic inflammation in the setting of a stable and contained GIH can result in the eventual resolution of the GIH without the need for invasive intervention. In summary, a GIH should be recognized as a rare consequence of pancreatitis (acute, necrotizing, hemorrhagic, etc.) and can be managed conservatively given the correct clinical scenario.
